# Prognostic Signature of Immune Genes and Immune-Related LncRNAs in Neuroblastoma: A Study Based on GEO and TARGET Datasets

**DOI:** 10.3389/fonc.2021.631546

**Published:** 2021-03-09

**Authors:** Xiaodan Zhong, Ying Tao, Jian Chang, Yutong Zhang, Hao Zhang, Linyu Wang, Yuanning Liu

**Affiliations:** ^1^ College of Computer Science and Technology, Jilin University, Changchun, China; ^2^ Department of Pediatric Oncology, The First Hospital of Jilin University, Changchun, China; ^3^ Key Laboratory of Symbolic Computation and Knowledge Engineering, Ministry of Education, Jilin University, Changchun, China; ^4^ Department of Anesthesiology, China-Japan Union Hospital of Jilin University, Changchun, China

**Keywords:** immune-related, neuroblastoma, prognosis, validation, lncRNAs

## Abstract

**Background:**

The prognostic value of immune-related genes and lncRNAs in neuroblastoma has not been elucidated, especially in subgroups with different outcomes. This study aimed to explore immune-related prognostic signatures.

**Materials and Methods:**

Immune-related prognostic genes and lncRNAs were identified by univariate Cox regression analysis in the training set. The top 20 C-index genes and 17 immune-related lncRNAs were included in prognostic model construction, and random forest and the Least Absolute Shrinkage and Selection Operator (LASSO) regression algorithms were employed to select features. The risk score model was constructed and assessed using the Kaplan-Meier plot and the receiver operating characteristic curve. Functional enrichment analysis of the immune-related lncRNAs was conducted using the STRING database.

**Results:**

In GSE49710, five immune genes (CDK4, PIK3R1, THRA, MAP2K2, and ULBP2) were included in the risk score five genes (RS5_G) signature, and eleven immune-related lncRNAs (LINC00260, FAM13A1OS, AGPAT4-IT1, DUBR, MIAT, TSC22D1-AS1, DANCR, MIR137HG, ERC2-IT1, LINC01184, LINC00667) were brought into risk score LncRNAs (RS_Lnc) signature. Patients were divided into high/low-risk score groups by the median. Overall survival and event/progression-free survival time were shortened in patients with high scores, both in training and validation cohorts. The same results were found in subgroups. In grouping ability assessment, the area under the curves (AUCs) in distinguishing different groups ranged from 0.737 to 0.94, better in discriminating MYCN status and high risk in training cohort (higher than 0.9). Multivariate Cox analysis demonstrated that RS5_G and RS_Lnc were the independent risk factors for overall and event/progression-free survival (all p-values <0.001). Correlation analysis showed that RS5_G and RS_Lnc were negatively associated with aDC, CD8+ T cells, but positively correlated with Th2 cells. Functional enrichment analyzes demonstrated that immune-related lncRNAs are mainly enriched in cancer-related pathways and immune-related pathways.

**Conclusion:**

We identified the immune-related prognostic signature RS5_G and RS_Lnc. The predicting and grouping ability is close to being even better than those reported in other studies, especially in subgroups. This study provided prognostic signatures that may help clinicians to choose optimal treatment strategies and showed a new insight for NB treatment. These results need further biological experiments and clinical validation.

## Introduction

Neuroblastoma (NB), one of the most common malignant solid tumors in children, accounts for nearly 15% of childhood cancer-related deaths (1, 2). At present, treatment strategy selection for NB is mainly based on risk stratification. Over the past two decades, the International Neuroblastoma Risk Group (INRG) classification system has defined a unified approach for pretreatment assessment, which includes age at diagnosis, histology, the grade of tumor differentiation, MYCN status, 11q aberration, and ploidy (3). According to the classification criteria, low- and intermediate-risk patients can receive reduced chemotherapy and attain good outcomes. However, the prognosis for high-risk patients is still dismal, even though they have received intensive chemotherapy, surgery, radiotherapy, and other combined therapies, and the long-term survival of these patients remains less than 50% (4–6). Besides, the clinical use of these markers may be limited in some patients despite the elaborate risk stratification.

Thanks to the development of microarrays and high-throughput technologies, an increasing number of abnormalities have been discovered in both driver and non-driver genes in NB (7). Unlike adult cancers, the total somatic mutation rate is less than 20%, even in the high risk NB, as reported by a study from TARGET enrolling 240 patients. Somatic mutations are most commonly seen in ALK (9.2%), followed by PTPN11 (2.9%), ATRX (2.5%), and MYCN (1.7%) (8). Typically, ARID1A and AIRD1B alterations have been identified through integrated genomic analysis, and they are correlated with the poor response to early treatment and unfavorable outcomes (2). Therefore, targeted mutation therapy is not suitable for tumors in children with low mutation frequency, such as NB. In this regard, it is still necessary and urgent to identify new molecular targets and to improve the prognostic outcomes in children with NB.

The tumor microenvironment (TME) has been confirmed to play an important role in various cancer types, including NB (4). The TME components, such as immunocytes (9) and stromal cells, participate in numerous cancer progresses, such as tumorigenesis, metastasis (10, 11), response to immune checkpoint blocking therapy (12), and prediction of clinical outcomes (13, 14). It is shown that the spontaneous regression in stage 4s NB may be associated with cellular immunity (15). Besides, some researchers have proved that immunocytes like tumor-associated macrophages (TAMs) and cancer-associated fibroblast (CAF) are markedly correlated with the unfavorable consequences in NB (16). Moreover, the impact of tumor-infiltrating immunocytes on the prediction of NB prognosis has also been examined (17, 18).

Liu Z depicted the immune gene expression profiles in high risk NB and found an ultra-high risk NB (UHR-NB) group that showed the worst prognosis. Besides, the authors identified four up-regulated immune genes (ADAM22, GAL, KLHL13, and TWISTT1) among the UHR-NB patients (19). However, to date, the prognostic value of immune-related genes and lncRNAs in NB has not been elucidated yet. The anti-GD2 monoclonal antibody is the only immune therapeutic agent used for high risk NB patients, which improves the 2-year overall survival (OS) and event-free survival (EFS). However, it is associated with drawbacks such as significant cumulative toxicity and its improvement of long-term survival is unclear (20). It is noteworthy that the low toxicity of anti-tumor agents and long-term survival are quite important for children.

The present study aimed to explore the prognostic immune-related genes and lncRNAs, and to discuss their feasibility as prognostic and follow-up markers in NB to help clinicians optimize individual treatment protocols. This study focused on the significance of immune-related prognostic signatures in predicting outcomes for NB patients in each group.

## Materials and Methods

### Data Processing

Neuroblastoma training set GSE49710 (n=498, GPL16876) was downloaded from the GEO database (https://www.ncbi.nlm.nih.gov/gds/?term=), validation cohorts were downloaded from R2 database (https://hgserver1.amc.nl/cgi-bin/r2/main.cgi), including Neuroblastoma public - Versteeg - 88 (GSE16476, GPL570), TARGET- Asgharzadeh - 249, and Neuroblastoma Primary - NRC - 283 (GSE85047, GPL5175). We excluded 5 patients with MYCN amplification status NA, and included 493 patients as a training cohort (n=493), the validation sets were GSE16476, TARGET – 249, and GSE85047.

If one gene corresponded to multiple probes, the highest expression value was taken as the representative and log2 transformed. For lncRNAs, we confirmed them as “ncRNAs” from the NCBI database (https://www.ncbi.nlm.nih.gov/gene/).

### Identification of Survival-Related Immune Genes

The immune-related gene list was downloaded from the ImmPort database (21) (https://immport.niaid.nih.gov/), including 1534 genes, after excluding duplicates and mismatches, a total of 993 genes remained. Univariate Cox proportional model survival analysis (22) was performed in GSE49710. Genes with p <0.01 were considered as survival-related immune genes.

### Construction of the Immune-Related Prognostic Signatures

Univariate Cox regression analysis was used to select candidate immune-related genes and rank them according to the Concordance index. The Random Forest algorithm (23, 24) was employed to identify the importance of the top 20 C-index immune-related genes in vital status. We then carried out multivariate Cox regression and obtained the coefficient of the genes. At last, a prognostic score model was constructed, including five genes (CDK4, PIK3R1, MAP2K2, ULBP2, and THRA).

$$Prognostic\;Score=\sum_{i=1}^{n}\beta i*xi$$

where *βi* represented the coefficient of each gene got from multivariate Cox regression analysis of overall survival time, and *xi* indicated the expression value by log2 transformed of the corresponding gene.

All patients had risk scores and were divided into high-risk and low-risk score groups according to the median value. The Kaplan-Meier curves of overall survival and event/progression/relapse-free survival were plotted.

### Extraction of Immune-Related LncRNAs and Construction of Prognostic lncRNA Signature

LncRNAs correlated with the five genes which Spearman Correlation Coefficient >0.5 as immune-related lncRNAs. We chose the Spearman correlation because of the non-normal distribution of the gene expression values. The Least Absolute Shrinkage and Selection Operator (LASSO) regression analysis (25) was performed to screen the prognostic immune-related lncRNA signature, and minimal lambda was considered optimal. The modeling process is the same as above.

### Functional Annotation and Enrichment Analysis

To explore the biological functions of prognostic immune genes and immune-related lncRNAs, KEGG (26) (Kyoto Encyclopedia of Genes and Genomes) in the STRING (27) database (https://string-db.org/) was performed. FDR <0.05 was considered significant.

### Cell Fraction Calculation in Tumor Microenvironment

To describe the cell composition of the tumor microenvironment (TME) in neuroblastoma, we employed the xCell algorithm (28) (http://xcell.ucsf.edu/). The xCell scores of cell types represented a fraction of cells in tumor tissues.

### Statistical Analysis

In this study, the R software version 3.6.2 and the GraphPad Prism version 8.0 were used. Univariate and multivariate Cox regression analyses were performed by R package “survival”, at the same time, Harrell’s concordance index (C-index) was calculated to assess the performance of the model. The ggforest plots were generated by R package “survminer”. Kaplan-Meier algorithm was used to evaluate the survival time of high/low-risk groups, and a Log-rank test was employed. To test the predictive ability of the prognostic signature, the area under the curves (AUC) of receiver operating characteristic (ROC) was calculated and plot by R package “pROC”. The random forest algorithm was carried out by R package “randomForest”. The Lasso regression analysis was implemented by R package “glmnet”. The Spearman Correlation Coefficient was calculated by R method “cor.test ()”, and the correlation heatmaps were plotted by GraphPad Prism 8.0. The Mann-Whitney U test was used to compare the xCell score of cells in different groups. All statistical tests were two-sided and a p-value < 0.05 was considered statistically significant.

## Results

### Identification of Survival-Related Immune Genes

Univariate Cox regression analysis was performed in the GSE49710 dataset. Altogether 681 immune-related prognostic genes associated with OS time were identified, and a p-value <0.01 indicated statistical significance. Among these identified genes, 597 were positively correlated whereas 84 were negatively correlated with survival. As suggested by functional enrichment analysis, these genes were mainly enriched in tumor-related pathways (like the PI3K-AKT, MAPK, Ras signaling pathways) and immune-related pathways (such as NK cell-mediated cytotoxicity and T cell receptor signaling pathway), as shown in **Supplementary Figure 1**. The top 20 prognostic immune-related genes in terms of their C-indexes were enrolled for subsequent model screening (**Table 1**).

**Table 1:** Top 20 prognostic immune-related genes in GSE49710.

Gene Symbol	HR	p-value	C-index	Chr	Gene Symbol	HR	p-value	C-index	Chr
PLXNA4	0.63	<2e-16	0.799	7	CDK4	1.837	<2e-16	0.756	12
THRA	0.397	<2e-16	0.785	17	GHRL	0.527	<2e-16	0.754	3
PLXNC1	0.662	<2e-16	0.785	12	MC4R	0.639	<2e-16	0.754	18
PIK3R1	0.51	<2e-16	0.782	5	PPP3CB	0.336	<2e-16	0.753	10
TNFRSF25	0.662	<2e-16	0.781	1	LIFR	0.555	<2e-16	0.752	5
UTS2D	0.512	<2e-16	0.769	3	SCG2	0.666	<2e-16	0.748	2
RORA	0.489	<2e-16	0.767	15	MIA	0.614	<2e-16	0.748	19
ULBP2	0.559	<2e-16	0.765	6	MAP2K2	3.80	1.7E-15	0.746	19
PIK3CD	0.537	<2e-16	0.761	1	NRG1	0.579	<2e-16	0.746	8
ADRB2	0.684	<2e-16	0.757	5	PPP3CC	0.354	<2e-16	0.742	8

### Feature Selection by Random Forest

**Figure 1:**
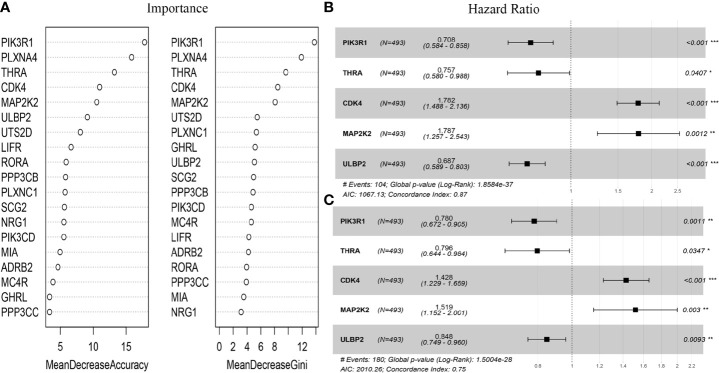
Immune genes in RS5_G. **(A)** Importance of top20 C-index immune genes. **(B, C)** Univariate Cox regression analysis for overall survival and event-free survival. *p<0.05, **p<0.01, ***p<0.001.

The immune-related genes in **Table 1** were included in the prognostic model feature importance selection. Meanwhile, the random forest algorithm was employed to select the important immune-related genes. The above-mentioned 20 genes were used as the features to discriminate the vital status, then the mean decrease accuracy (MDA) and mean decrease Gini (MDG) were obtained. After repeating the algorithm 100 times, the average out-of-bag (OOB) value was 16.99%. As shown in **Figure 1A**, for the five genes incorporated into the prognostic score model, their importance values were among the top six of those top 20 gene rankings. Thereafter, their coefficients were obtained through multivariate Cox regression analysis, according to the following formula: Prognostic Score = (-0.34546*PIK3R1) + (-0.27783*THRA) + (0.57802*CDK4) + (0.58081*MAP2K2) + (-0.3748*ULBP2). Results of univariate Cox regression analysis for the five genes are shown in **Figures 1B, C**. Then, the immune-related gene combination was named prognostic model RS5_G.

### Prognostic Model RS5_G Had Good Performance in Predicting Neuroblastoma Outcomes

**Figure 2:**
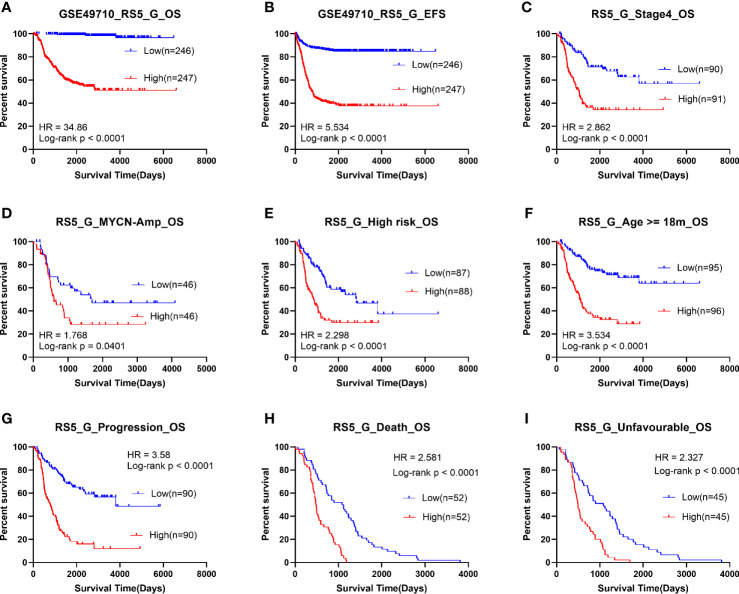
Survival analysis of RS5_G in GSE49710. **(A, B)** Overall/event-free survival of high/low-risk socre group. **(C-I)** Overall survival of patients with high/low risk scores in subgroups.

Patients in the GSE49710 dataset (training cohort) were divided into high- or low-risk score groups according to the median RS5_G prognostic score. As shown in **Figure 2**, the OS time and EFS time of the high risk group were greatly inferior to those of low risk group. To assess the predictive performance, stratification survival analysis was performed in clinical subgroups. In unfavorable clinical subgroups (including stage 4, MYCN amplified, high risk, age ≥ 18 months, progression, and class label unfavorable groups), the OS time of patients with high prognostic scores was significantly worse (all p-values <0.0001).

**Figure 3:**
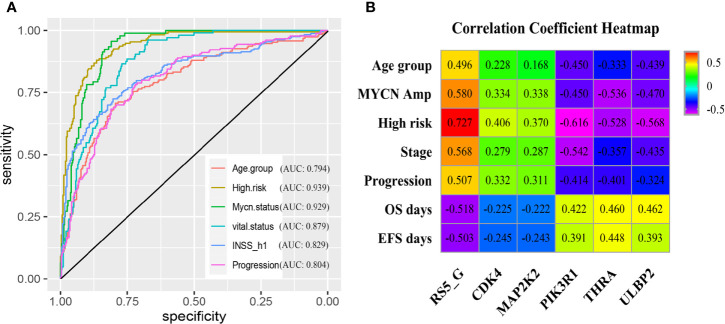
Clinical performance of RS5_G in GSE49710. **(A)** ROC curve of RS5_G in grouping. **(B)** Spearman correlation of RS5_G and the immune genes with clinical features.

Moreover, the grouping ability of the prognostic signature was also evaluated by the receiver operating characteristic (ROC) curve analysis. As a result, the area under the curve (AUC) values of age group, high risk, MYCN status, vital status, INSS_h1and progression were 0.794 (0.752-0.836), 0.939 (0.918-0.96), 0.929 (0.907-0.952), 0.879 (0.848-0.91), 0.829(0.792-0.865) and 0.804 (0.764-0.845), respectively (**Figure 3A**). To verify the associations between RS5_G (including the immune genes) and clinical characteristics, their corresponding Spearman correlation coefficient (SCC) was calculated, as shown in **Figure 3B**. The results showed that RS5_G, CDK4, and MAP2K2 are positively correlated to unfavorable prognostic factors, including age group, MYCN amplified, INRG high risk, INSS stage, and progression, which were negatively correlated to OS and EFS days, whereas the expression of PIK3R1, THRA, and ULBP2 showed the opposite results.

The following parameters were incorporated in both univariate and multivariate Cox regression analyses, including RS5_G, age group, high risk, MYCN status, and INSS_h1 (0 for stage 1/2/4s, 1 for stage 3/4). The results showed that the above-mentioned features were all risk factors. The _G high, age ≥18 months, high risk, MYCN amplified, and INSS stage 3/4 were correlated with unfavorable outcomes. However, the RS5_G signature had the highest C-index (**Table 2**). Upon multivariate Cox analysis, RS5_G and high risk were identified as the independent prognostic factors for OS, whereas RS5_G, high risk, and INSS_h1 were the independent prognostic factors for EFS. However, RS5_G had the most significant p-value (**Table 3**). We then compared the above risk factors with RS5_G seriatim, the results revealed that RS5_G is the only independent prognostic risk factor (**Supplementary Table 1**).

**Table 2:** Univariate Cox regression analysis in GSE49710.

Features	Overall Survival				Event-free Survival			
	HR	95% CI	p-value	C-index	HR	95% CI	p-value	C-index
RS5_G	2.727	2.348-3.167	<2E-16	0.869	1.893	1.708-2.097	<2E-16	0.742
Age group	8.497	5.211-13.86	<2E-16	0.727	3.339	2.462-4.529	9.06E-15	0.628
High risk	20.84	11.6-37.43	<2E-16	0.806	5.302	3.875-7.254	<2E-16	0.679
Mycn status	7.651	5.167-11.33	<2E-16	0.705	3.231	2.36-4.425	2.58E-13	0.598
INSS_h1	14.35	7.238-28.45	2.38E-14	0.742	4.68	3.297-6.642	<2E-16	0.663
RS_Lnc	2.718	2.331-3.17	<2E-16	0.851	1.85	1.658-2.065	<2E-16	0.709

**Table 3:** Multivariate Cox regression analysis in GSE49710.

Features	Overall Survival				Event-free Survival			
	HR	95% CI	p-value	C-index	HR	95% CI	p-value	C-index
RS5_G	1.874	1.504-2.335	2.15E-08	0.881	1.626	1.385-1.98	2.65E-09	0.751
Age group	1.134	0.592-2.171	0.704		0.95	0.602-1.499	0.826	
High risk	3.804	1.604-9.024	0.002		1.82	1.026-3.23	0.041	
Mycn status	1.102	0.679-1.789	0.695		0.712	0.468-1.082	0.11	
INSS_h1	2.302	0.99-5.352	0.05		1.788	1.124-2.845	0.014	

### Immune-Related Prognostic lncRNA Signature Performed Well in Predicting Outcomes and Grouping Different Prognostic Groups of Neuroblastoma

The SCCs were calculated between lncRNAs and genes in the RS5_G signature. The lncRNAs with SCC >0.5 and univariate Cox regression p-value <0.01 were selected, as the immune-related lncRNAs. Finally, 17 lncRNAs were identified, as presented in **Supplementary Figure 2A**. At the same time, the Lasso regression algorithm was employed to select the important lncRNAs for the discrimination of vital status. As illustrated from **Supplementary Figure 2B**, the optimal model with the lowest lambda value contained eleven lncRNAs, including LINC00260, FAM13A1OS, AGPAT4-IT1, DUBR, MIAT, TSC22D1-AS1, DANCR, MIR137HG, ERC2-IT1, LINC01184, and LINC00667. These lncRNAs were differentially expressed in diverse the prognostic groups mentioned above (**Supplementary Figure 3**). Similarly, the coefficients of these eleven lncRNAs were obtained according to the above-mentioned method: Prognostic Score = (-0.08876*LINC00260) + (-0.68471*FAM13A1OS) + (-0.40775*AGPAT4-IT1) + (-0.63961*DUBR) + (0.0409*MIAT) + (-0.1759*TSC22D1-AS1) + (0.60318*DANCR) + (-0.04458*MIR137HG) + (0.63221*ERC2-IT1) + (0.41124*LINC01184) + (0.17513*LINC00667). The prognostic signature of immune-related lncRNAs was then called RS_Lnc. Results of Lasso Cox survival analysis for lncRNAs in GSE49710 are displayed in **Supplementary Figure 2**. The nomogram of the eleven lncRNAs is shown in **Supplementary Figure 2C, D**.

**Figure 4:**
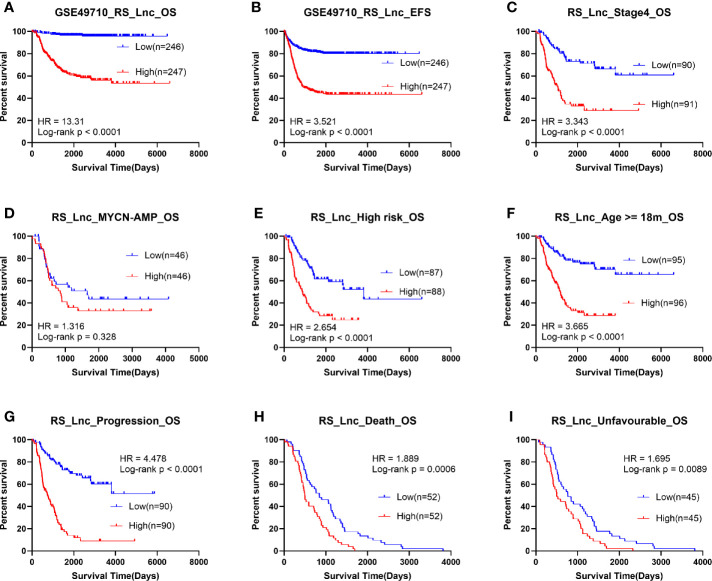
Survival analysis of RS_Lnc in GSE49710. **(A, B)** Overall/event-free survival of high/low-risk score group. **(C-I)** Overall survival of patients with high/low risk scores in subgroups.

Survival analysis was subsequently implemented for RS_Lnc, and the results showed that patients with high scores had a significantly shorter OS and EFS time than those with low scores. The results of subgroup analysis showed that the OS time of patients in high risk groups for all subgroups was significantly shortened, except for MYCN amplified groups (**Figure 4**).

**Figure 5:**
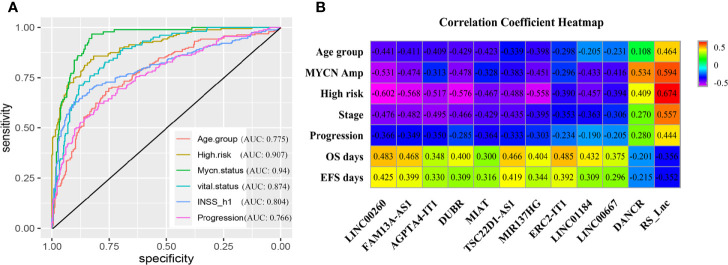
Clinical performance of RS_Lnc in GSE49710. **(A)** ROC curve of RS_Lnc in grouping. **(B)** Spearman correlation of RS_Lnc and immune-related lncRNAs with clinical features.

Thereafter, the ROC curve analysis was adopted to assess performance in discriminating different prognostic groups by RS_Lnc. As a result, the AUC values of age group, high risk, MYCN status, vital status, INSS_h1 and progression were 0.775 (0.732-0.818), 0.907 (0.879-0.934), 0.94 (0.918-0.962), 0.874 (0.84-0.909), 0.804 (0.764-0.844) and 0.766 (0.722-0.811), respectively (**Figure 5A**). To confirm the associations of RS_Lnc (including the immune-related lncRNAs) with clinical characteristics, their corresponding SCCs were calculated, as shown in **Figure 5B**. The results indicated that RS_Lnc and DANCR are positively correlated to inferior prognostic factors, including age >18 months, MYCN amplified, INRG high risk, advanced stage, and progression, which were negatively correlated to OS and EFS days, whereas the expression of the other ten lncRNAs displayed the opposite results.

Upon univariate Cox analysis, RS_Lnc was associated with short OS and EFS time (HR: 2.718, 1.85; p-value: < 2E-16). Multivariate Cox analysis identified that RS_Lnc and high risk were the independent prognostic factors for OS, whereas RS_Lnc, high risk, and INSS_h1 were the independent prognostic factors for EFS. However, RS_Lnc had the most significant p-value (**Supplementary Table 2**).

### Immune-Related lncRNAs Mainly Enriched in Cancer- and Immune-Related Pathways

**Figure 6:**
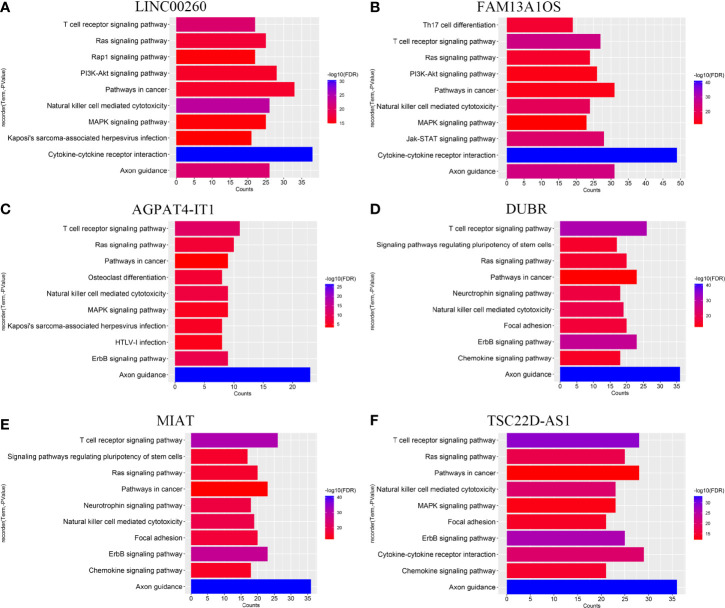
KEGG pathway of **(A)** LINC00260, **(B)** FAM13A1OS, **(C)** AGPAT4-IT1, **(D)** DUBR, **(E)** MIAT, and **(F)** TSC22D-AS1.

To explore the biological functions of immune-related lncRNAs in the prognostic signature, genes with lncRNAs SCCs > 0.5 were screened and the online database STRING was utilized to obtain the protein-protein interaction (PPI) network. The KEGG pathways are shown in **Figure 6** and **Supplementary Figure 4**. These lncRNAs were mainly enriched in some molecular signaling pathways (such as PI3K-AKT, Ras, MAPK, and ErbB signaling pathways) and some immune-related pathways (like T cell receptor signaling pathway and natural killer cell-mediated cytotoxicity).

### Immune Infiltration and Correlation With Prognostic Signature

**Figure 7:**
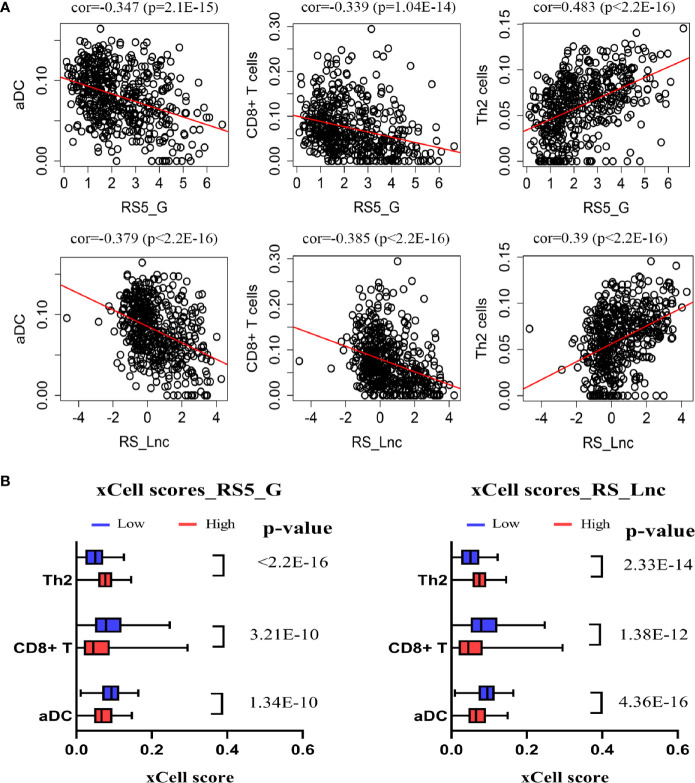
Correlation of prognostic signature and immune cells. **(A)** Spearman correlation of signature and immune cells. **(B)** xCell scores of immune cells in high/low-risk score groups.

In this study, the xCell algorithm was employed to calculate the xCell scores of different cell types in the tumor tissues of NB patients from the GSE49710 dataset, which could represent the proportions of various cells in the tumor microenvironment (TME). Typically, the activated dendritic cells (aDC), T helper cells (Th2 cells), and CD8+ T cells, and their correlations with the prognostic signatures RS5_G and RS_Lnc were analyzed, respectively. These three cell types were chosen because of their higher xCell scores and their associations with tumor prognosis. As shown in **Figure 7**, aDC and CD8+ T cells were negatively correlated with RS5_G and RS_Lnc, whereas Th2 cells were positively correlated with the above two prognostic signatures. There were significant differences in these three cell types between the high- and low-risk score groups.

### Performance Validation in the Independent Datasets

To further confirm the robustness of the prognostic signature RS5_G, its performance was validated in another three independent datasets, GSE16476, TARGET- Asgharzadeh – 249, and GSE85047. Furthermore, the performance of RS_Lnc was also validated in GSE16476.

**Figure 8:**
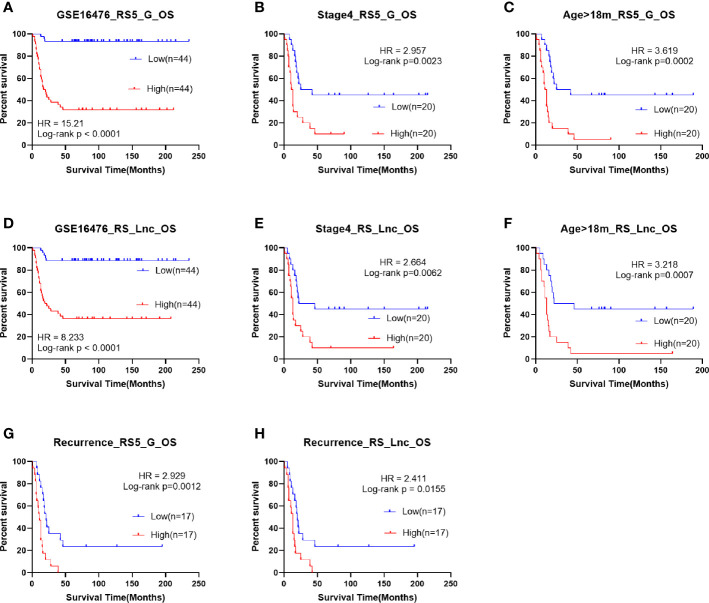
Survival analysis of RS5_G and RS_Lnc in GSE16476. **(A-C, G)** Overall survival of RS5_G high/low-risk score group. **(D-F, H)** Overall survival of RS_Lnc high/low risk score group.

**Figure 9:**
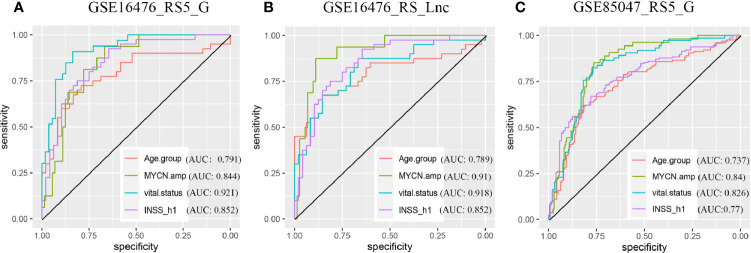
ROC in validation datasets **(A, B)** ROC curves of RS5_G and RS_Lnc in grouping in GSE16476. **(C)** ROC curve of RS5_G in grouping in GSE85047.

The OS and progression-free survival (PFS) time were significantly shorter in high-risk score groups than in low-risk groups of the GSE16476 dataset, both for RS5_G and RS_Lnc prognostic signatures, as shown in **Figure 8**. Subgroup analyses also came to similar results (all p-values < 0.05). It was found that these two prognostic signatures had satisfactory performance in distinguishing different prognostic groups. Typically, the AUC values of age group, MYCN amplified, vital status, and INSS_h1 reached 0.791 (0.689-0.892), 0.844 (0.756-0.932), 0.921 (0.866-0.976) and 0.852 (0.771-0.932), respectively, for RS5_G signature. As for RS_Lnc, the AUC values were 0.789 (0.686-0.892), 0.91 (0.839-0.981), 0.918 (0.861-0.975) and 0.852 (0.771-0.932), respectively (**Figures 9A, B**).

Univariate Cox regression analysis identified that RS5_G and RS_Lnc were the risk factors for OS time in GSE16476, which had higher C-indexes than the other risk factors (age group, MYCN amplified, and INSS_h1) (**Supplementary Table 3**). Upon multivariate Cox analysis, RS5_G, RS_Lnc, and age group were identified as independent risk factors. However, our signature had a more significant p-value (**Supplementary Table 4**).

In the TARGET-249 and GSE85047 cohorts, some immune-related lncRNAs were not detected. Therefore, the robustness of the RS5_G signature was validated. In TARGET-249, two patients with no available survival information were excluded, and finally, 247 patients were included in subsequent analysis. The results suggested that patients with high-risk scores had inferior outcomes to those with low-risk scores. In subgroups analysis including INSS stage 4, event, high risk, age ≥18 months, pathological diagnosis as NB, undifferentiated or poorly differentiated, and unfavorable histology, patients with high-risk scores had visibly reduced OS time (**Supplementary Figure 5**). In the GSE85047 dataset, a total of 268 patients were included. It was found that OS and PFS were significantly shortened in high-risk score groups (**Supplementary Figure 6**). Subgroup analysis also came to consistent results. RS5_G had the highest C-indexes in univariate Cox regression analysis of OS and PFS (**Supplementary Table 5**). Moreover, results of multivariate Cox analysis displayed that, RS5_G and INSS_h1 were the independent risk factors; however, RS5_G had a more significant p-value (**Supplementary Table 6**). ROC curves analysis revealed that the AUC values of RS5_G in discriminating age group, MYCN amplified, vital status and INSS_h1 were 0.737 (0.676-0.798), 0.84 (0.788-0.892), 0.826 (0.773-0.879) and 0.77 (0.713-0.827), respectively (**Figure 9C**).

## Discussion

Neuroblastoma (NB) is a malignant solid tumor in children, which is linked with highly heterogeneous outcomes ranging from spontaneous regression without treatment to rapid progression after intensive multidisciplinary therapy (3, 29). NB accounts for 8-10% of all malignancies in children and is responsible for 15% of pediatric cancer-related deaths (4, 5, 19, 30). Within the past few decades, the MYCN oncogene has been extensively and deeply studied, and more molecules have also been discovered to play important roles in tumor development, progression, and metastasis, including ALK (8), LMO1 (31, 32), LIN28B (33, 34), AURKA (35, 36), AIRD1A and ARID1B (2), etc. Besides, some gene inhibitors have been attempted to treat NB, like ALK and AURKA. Nevertheless, the long-term survival of NB at stage 4 or high risk has not been significantly improved yet, and there are still many relapsed or refractory patients. Hence, it is necessary to identify the novel molecules as the therapeutic targets or prognostic biomarkers, especially for high risk patients.

At present, NB treatment is mainly based on the INRG risk stratification system (3). Researchers have discovered some prognostic markers that may assist clinicians in choosing the optimal treatment regimen, but few patients benefit from these markers, especially those in subgroups (37–40). It has not yet been elucidated whether the prognosis of NB patients can be predicted by immune-related genes and immune-related lncRNAs and their correlations with clinicopathological characteristics. In this study, it was found that over 1/8 immune-related genes were positively correlated with NB survival, which meant that the expression of these genes was down-regulated in patients with poor prognosis, indicating that patients with poor prognosis might be in an immunosuppression state. Two immune-related prognostic signatures RS5_G and RS_Lnc were identified, and their predicting and grouping abilities were analyzed in another three independent datasets. The associations of these two signatures with clinicopathological features and some immunocytes were also examined.

Five genes, including CDK4, PIK3R1, THRA, MAP2K2, and ULBP2, were included in the immune-related gene signature. The use of CDK4 inhibitor alone or in combination with other drugs in the treatment of NB has entered a phase I clinical trial (41–43). MAP2K2, also referred to as MEK2, is an important molecule involved in the MAPK signaling pathway. The MEK inhibitor binimetinib combined with the CDK4/6 inhibitor ribociclib achieved a synergistic therapeutic effect in the preclinical model of high risk NB (42). In this study, CDK4 and MAP2K2 were negatively correlated with long-term survival time. PIK3R1, which encodes the p85α (the regulatory subunit of PI3K), and plays an important role in the PI3K-AKT signaling pathway (44). Fulda elaborated on the feasibility of using the PI3K-AKT-mTOR pathway as a therapeutic target in NB (45). In our study, PIK3R1 was positively correlated with OS and EFS, which is consistent with its function. The above results demonstrated the accuracy and effectiveness of our prognostic gene screening method.

THRA is a gene encoding the specific high-affinity nuclear receptor that mediates thyroid hormone. Studies have shown that THRA and THRB (another gene that encodes this receptor), may be involved in human cancer (46). For instance, a study from Xiang Z et al. demonstrated that in ERBB2+ gastric cancer (GC), THRA might be one of the sensitive biomarkers for the targeted ERBB2 treatment, and its expression level was negatively correlated with Myc activation (47). In this study, it was discovered that patients with high THRA expression displayed favorable outcomes. NKG2D is an activating immune receptor found in CD8+ T cells and NK cells, and its ligands include ULBPs, MICA, and MICB. Raffaghello et al. found through experiments, that ULBP2 was expressed in half of primary tumors, and concluded that NB evaded immune cell killing by down-regulating and/or releasing the NKG2D ligands (48). In our study, patients with poor prognosis had reduced ULBP2 expression, and high ULBP2 expression was associated with longer OS and EFS time.

The RS5_G signature constructed in this study performed well in predicting prognosis, both in training and validation cohorts, especially in subgroups. Taking the performance of RS5_G in distinguishing the survival differences in INRG high risk group as an example, our signature achieved comparable results with fewer genes compared with previous studies. Liu identified an ultra-high risk NB (UHR-NB) group by employing the spectral co-clustering algorithm based on 283 immune-related genes and the two-year survival rates were 48.8% and 43.2% for TARGET and GSE49710, respectively; whereas the two-year survival rates were 79% and 75% in HR-NB group, respectively (19). In our study, the high risk patients were divided into high or low risk score groups according to the median. In the high-risk score groups, the two-, three- and five-year survival rates were 58.7%, 45.0% and 33.9% in TARGET; while 58.0%, 43.2% and 37.5% in GSE49710, respectively. In low-risk score groups, the two-, three-, and five-year survival rates were 78.7%, 64.8%, and 50.9% in TARGET, whereas 83.9%, 78.2%, and 64.4% in GSE49710, respectively. The two-year survival rates of our groups were higher than Liu Z for the unfavorable prognostic groups, yet, only five genes were used in our study, which achieved higher feasibility in clinical trials. Notably, the survival time was distinctly different between high- and low-risk score groups. Besides, the genes included in our signature are different from those previously published by Liu et al, we considered that it is due to our different methods and concerns. By comparing acute lymphoblastic leukemia (ALL), acute myeloid leukemia (AML), Wilm’s tumor (WT), and NB, Liu et al. screened out NB-specific immune-related genes and paid attention to their relationship with prognosis, whereas we focused on immune-related genes that are closely related to NB survival. However, the two signatures screened by us and Liu et al. screen out UHR-NB patients well. Moreover, the robustness of the RS5_G signature was also validated in another two independent cohorts, GSE16476 and GSE85047, and the results were satisfying.

The results of multivariate Cox regression analysis indicated that RS5_G, high risk, and INSS_h1 can be independent prognostic factors for OS and EFS in GSE49710, and RS5_G had the most significant p-value. It demonstrated that the RS5_G signature might help clinicians to choose the appropriate therapeutic regimen for different patients. For example, we can choose a stronger plan at the time of initial treatment for patients with high-risk scores, rather than the intensive treatment at the time of relapse. By comparing RS5_G with the characteristics previously included in NB risk stratification through multivariate Cox analysis, we believe that the immune-related prognostic signature screened by gene expression profiles can help clinicians distinguish patients with different outcomes in INRG high risk groups, to give different treatment regimens. Of course, further experimental verification is needed for accurate application in clinical practice, in particular, the cut-off value is needed to confirm by moderate methods.

Long non-coding RNAs (lncRNAs) participate in the pathological process of tumors including NB in a variety of ways (6, 49), and lncRNAs may serve as the prognostic markers for various cancers (37). For example, NBAT1 is located on chromosome 6p22, regulates NB progression, and is correlated with the favorable outcome of NB (50). In addition, LncNB1 plays an oncogenic role in NB tumorigenesis (51). Gao L constructed a risk signature comprised of 10 lncRNAs, with the AUC value of as high as 0.941 in predicting patients’ survival (37). However, its generalization performance was unsatisfactory and the signature was not further verified in more independent datasets. In this study, an immune-related lncRNA prognostic signature incorporating 11 lncRNAs was constructed and called RS_Lnc. It served as an independent prognostic factor for OS and EFS in GSE49710 and OS in GSE16476 and had the most significant p-value. Gao identified 10 lncRNAs through differentially expressed analysis and incorporate them in a prognostic signature (37). By contrast, the 11 lncRNAs identified in this study were not the same as the results of the study by Gao, which might be ascribed to the different dataset detection and screening methods.

These eleven lncRNAs were associated with good OS and EFS, except for DNACR that was related to adverse outcomes. Few of them are correlated with cancer tumorigenesis and metastasis. For example, DANCR has been proven to participate in several cancer pathological processes, including tumor cell proliferation, invasion, metastasis, and chemo-resistance, and it may serve as a therapeutic target (52, 53). FAM13A1OS (FAM13A-AS1) has been discovered to show high expression in low risk NB patients, which is associated with autophagy and long-term survival (54). Furthermore, DUBR has also been reported to be down-regulated in high risk NB and differentially expressed between favorable and unfavorable outcomes (55). MIAT promotes the growth and metastasis of GC and colorectal cancer (CRC) cells *via* different pathways (56, 57), and affects the response of breast cancer (BC) cells to estrogen (58). LINC00667 can promote cell proliferation and migration in non-small cell lung cancer (NSCLC) (59), CRC (60), and Wilm’s tumor (61). However, in this study, MIAT and LINC00667 were correlated with favorable outcomes, and the functions of MIAT in NB should be further confirmed in biological experiments. MiR137 plays a role as a tumor suppressor in cancer; meanwhile, HSF1 can directly interact with its host gene MIR137HG to inhibit its expression and promote CRC occurrence (62).

In our study, functional enrichment analysis showed that lncRNA-associated genes are mainly enriched in the cancer-related signaling pathways (like PI3K-AKT, Ras, MAPK signaling pathways) and immune-related pathways (such as the chemokine and T cell receptor signaling pathways, NK cell-mediated cytotoxicity pathway). This indicates that in-depth research from the aspects of signaling pathways and immune response can be considered in the treatment of NB.

Immunocytes, including CD8+ T cells, NK cells, Th2 cells, etc., may assist patients in killing cancer cells or help tumor cells to evade immune killing (11, 20). In our previous study, we discovered that CD8+ T cells and aDCs are correlated with favorable outcomes, whereas Th2 cells are associated with unfavorable survival in NB (63). Fruci and colleagues have reported that intratumoral DCs and NK cells are associated with increased T-cell infiltration and favorable survival of NB, and identified two immune gene signatures associated with DC and NK cells that can predict the prognosis of NB patients (64). In this work, the xCell algorithm was employed to mimic immune cell proportion, and the correlations of Th2 cells, CD8+ T cells, and aDCs with the prognostic signature were calculated. It was found that the risk scores were remarkably negatively correlated with CD8+ T cells and aDCs, but positively associated with Th2 cells. These results demonstrated that both innate immunity and acquired immunity may play important roles in NB. The correlations of RS5_G and RS_Lnc with clinicopathological features were examined. As a result, these two signatures were positively correlated with known risk factors of NB but negatively associated with OS and EFS time.

Moreover, the grouping ability of our signature was verified in three datasets, GSE49710, GSE16476, and GSE85047. The AUC values in distinguishing different groups ranged from 0.737 to 0.94. Typically, the signature performed well in discriminating MYCN status and vital status compared with other groups. In the future, further research is warranted to examine the relationship between genes/lncRNAs and MYCN amplified and to explore the possibility of these genes as therapeutic targets.

Even though our prognostic signatures performed well in both the training set and validation cohorts, certain limitations should be noted in this study. Firstly, the datasets came from different platforms, and there were differences in testing, which might cause some bias in the results. Secondly, some lncRNAs were not tested in all datasets, and the performance of the RS_Lnc signature was not verified in all validation cohorts. Thirdly, our study was based on data mining, and these findings should be further validated through biological experiments and clinical validations.

Despite these shortcomings, the RS5_G and RS_Lnc signatures constructed in this study performed well at predicting prognosis and grouping different prognostic groups. In conclusion, an immune-related prognostic signature incorporating fewer genes was constructed in this study, which has comparable and even better at predicting and grouping abilities than those reported in other studies. Some immune-related lncRNAs are found to be correlated with the survival of NB patients, which are used to build the prognostic signature RS_Lnc, and it achieved good robustness. Collectively, this study not only provides prognostic signatures to help clinicians to choose the optimal therapeutic strategies but also sheds new light on research on NB treatment.

## Data Availability Statement

Publicly available datasets were analyzed in this study. This data can be found here: The expression data of training cohort GSE49710 (n=498, GPL16876) was downloaded from GEO database (https://www.ncbi.nlm.nih.gov/gds/?term=), survival information and validation datasets were downloaded from R2 database (https://hgserver1.amc.nl/cgi-bin/r2/main.cgi), including Neuroblastoma public—Versteeg—88 (GSE16476, GPL570), TARGET—Asgharzadeh—249, and Neuroblastoma Primary—NRC—283 (GSE85047, GPL5175).

## Author Contributions

YL conceived of and directed the project. XZ designed the study, analyzed the data, and wrote the first draft of the manuscript. YT and HZ collected data and samples and prepared figures and tables. YZ and JC revised the manuscript critically for important intellectual content. LW reviewed the data and corrected the algorithm. All authors have read and approved the final draft for publication. All authors contributed to the article and approved the submitted version.

## Funding

This study was funded by the Industrial Innovation Special Fund of Jilin Province (Grant Nos. 2019C053-2 and 2019C053-6) and the national key research and development program (Grant No. [2020]151).

## Conflict of Interest

The authors declare that the research was conducted in the absence of any commercial or financial relationships that could be construed as a potential conflict of interest.

## References

[1] Domingo-Fernandez R Watters K Piskareva O Stallings RL Bray I. The role of genetic and epigenetic alterations in neuroblastoma disease pathogenesis. Pediatr Surg Int (2013) 29(2):101–19. 10.1007/s00383-012-3239-7 PMC3557462 23274701

[2] Sausen M Leary RJ Jones S Wu J Reynolds CP Liu X. Integrated genomic analyses identify ARID1A and ARID1B alterations in the childhood cancer neuroblastoma. Nat Genet (2013) 45(1):12–7. 10.1038/ng.2493 PMC3557959 23202128

[3] Pinto NR Applebaum MA Volchenboum SL Matthay KK London WB Ambros PF. Advances in Risk Classification and Treatment Strategies for Neuroblastoma. J Clin Oncol (2015) 33(27):3008–17. 10.1200/JCO.2014.59.4648 PMC4567703 26304901

[4] Borriello L Seeger RC Asgharzadeh S DeClerck YA. More than the genes, the tumor microenvironment in neuroblastoma. Cancer Lett (2016) 380(1):304–14. 10.1016/j.canlet.2015.11.017 PMC5558454 26597947

[5] Ahmed AA Zhang L Reddivalla N Hetherington M. Neuroblastoma in children: Update on clinicopathologic and genetic prognostic factors. Pediatr Hematol Oncol (2017) 34(3):165–85. 10.1080/08880018.2017.1330375 28662353

[6] Salazar BM Balczewski EA Ung CY Zhu S. Neuroblastoma, a Paradigm for Big Data Science in Pediatric Oncology. Int J Mol Sci (2016) 18(1):1–27. 10.3390/ijms18010037 PMC5297672 28035989

[7] Huang CT Hsieh CH Lee WC Liu YL Yang TS Hsu WM. Therapeutic Targeting of Non-oncogene Dependencies in High-risk Neuroblastoma. Clin Cancer Res (2019) 25(13):4063–78. 10.1158/1078-0432.CCR-18-4117 30952635

[8] Pugh TJ Morozova O Attiyeh EF Asgharzadeh S Wei JS Auclair D. The genetic landscape of high-risk neuroblastoma. Nat Genet (2013) 45(3):279–84. 10.1038/ng.2529 PMC3682833 23334666

[9] Gajewski TF Schreiber H Fu YX. Innate and adaptive immune cells in the tumor microenvironment. Nat Immunol (2013) 14(10):1014–22. 10.1038/ni.2703 PMC4118725 24048123

[10] Hanahan D Coussens LM. Accessories to the crime: functions of cells recruited to the tumor microenvironment. Cancer Cell (2012) 21(3):309–22. 10.1016/j.ccr.2012.02.022 22439926

[11] Stolk D van der Vliet HJ de Gruijl TD van Kooyk Y Exley MA. Positive & Negative Roles of Innate Effector Cells in Controlling Cancer Progression. Front Immunol (2018) 9:1990. 10.3389/fimmu.2018.01990 30298063 PMC6161645

[12] Taube JM Galon J Sholl LM Rodig SJ Cottrell TR Giraldo NA. Implications of the tumor immune microenvironment for staging and therapeutics. Mod Pathol (2018) 31(2):214–34. 10.1038/modpathol.2017.156 PMC6132263 29192647

[13] Hui L Chen Y. Tumor microenvironment: Sanctuary of the devil. Cancer Lett (2015) 368(1):7–13. 10.1016/j.canlet.2015.07.039 26276713

[14] Jacquelot N Pitt JM Enot DP Roberti MP Duong CPM Rusakiewicz S. Immune biomarkers for prognosis and prediction of responses to immune checkpoint blockade in cutaneous melanoma. Oncoimmunology (2017) 6(8):e1299303. 10.1080/2162402X.2017.1299303 28919986 PMC5593705

[15] Brodeur GM Bagatell R. Mechanisms of neuroblastoma regression. Nat Rev Clin Oncol (2014) 11(12):704–13. 10.1038/nrclinonc.2014.168 PMC4244231 25331179

[16] Hashimoto O Yoshida M Koma Y Yanai T Hasegawa D Kosaka Y. Collaboration of cancer-associated fibroblasts and tumour-associated macrophages for neuroblastoma development. J Pathol (2016) 240(2):211–23. 10.1002/path.4769 PMC5095779 27425378

[17] Wei JS Kuznetsov IB Zhang S Song YK Asgharzadeh S Sindiri S. Clinically Relevant Cytotoxic Immune Cell Signatures and Clonal Expansion of T-Cell Receptors in High-Risk MYCN-Not-Amplified Human Neuroblastoma. Clin Cancer Res (2018) 24(22):5673–84. 10.1158/1078-0432.CCR-18-0599 PMC6504934 29784674

[18] Zhang P Wu X Basu M Dong C Zheng P Liu Y. MYCN Amplification Is Associated with Repressed Cellular Immunity in Neuroblastoma: An In Silico Immunological Analysis of TARGET Database. Front Immunol (2017) 8:1473. 10.3389/fimmu.2017.01473 29163537 PMC5675839

[19] Liu Z Grant CN Sun L Miller BA Spiegelman VS Wang HG. Expression Patterns of Immune Genes Reveal Heterogeneous Subtypes of High-Risk Neuroblastoma. Cancers (Basel) (2020) 12(7):1–16. 10.3390/cancers12071739 PMC7408437 32629858

[20] Szanto CL Cornel AM Vijver SV Nierkens S. Monitoring Immune Responses in Neuroblastoma Patients during Therapy. Cancers (Basel) (2020) 12(2):1–20. 10.3390/cancers12020519 PMC7072382 32102342

[21] Bhattacharya S Dunn P Thomas CG Smith B Schaefer H Chen J. ImmPort, toward repurposing of open access immunological assay data for translational and clinical research. Sci Data (2018) 5:180015. 10.1038/sdata.2018.15 29485622 PMC5827693

[22] Zhang Z Reinikainen J Adeleke KA Pieterse ME Groothuis-Oudshoorn CGM. Time-varying covariates and coefficients in Cox regression models. Ann Transl Med (2018) 6(7):121. 10.21037/atm.2018.02.12 29955581 PMC6015946

[23] Strobl C Boulesteix A-L Zeileis A Hothorn T. Bias in random forest variable importance measures: Illustrations, sources and a solution. BMC Bioinf (2007) 8:25. 10.1186/1471-2105-8-25 PMC1796903 17254353

[24] Wang H Yang F Luo Z. An experimental study of the intrinsic stability of random forest variable importance measures. BMC Bioinf (2016) 17:60. 10.1186/s12859-016-0900-5 PMC4739337 26842629

[25] Friedman J Hastie T Tibshirani R. Regularization Paths for Generalized Linear Models via Coordinate Descent. J Stat Software (2010) 33:1–22. 10.18637/jss.v033.i01 PMC2929880 20808728

[26] Kanehisa M Furumichi M Mao T Sato Y Morishima KJNAR. KEGG: new perspectives on genomes, pathways, diseases and drugs. Nucleic Acids Res (2017) 45: (Database issue):D353–D61. 10.1093/nar/gkw1092 PMC5210567 27899662

[27] Szklarczyk D Gable AL Lyon D Junge A Wyder S Huerta-Cepas J. STRING v11: protein-protein association networks with increased coverage, supporting functional discovery in genome-wide experimental datasets. Nucleic Acids Res (2019) 47(D1):D607–D13. 10.1093/nar/gky1131 PMC6323986 30476243

[28] Aran D Hu Z Butte AJ. xCell: digitally portraying the tissue cellular heterogeneity landscape. Genome Biol (2017) 18(1):220. 10.1186/s13059-017-1349-1 29141660 PMC5688663

[29] Nakagawara A Li Y Izumi H Muramori K Inada H Nishi M. Neuroblastoma. Jpn J Clin Oncol (2018) 48(3):214–41. 10.1093/jjco/hyx176 29378002

[30] Mullassery D Losty PD. Neuroblastoma. Paediatrics Child Health (2016) 26(2):68–72. 10.1016/j.paed.2015.11.005

[31] Liu Z Thiele CJ. When LMO, Meets MYCN, Neuroblastoma Is Metastatic. Cancer Cell (2017) 32(3):273–5. 10.1016/j.ccell.2017.08.014 PMC6294297 28898690

[32] Wang K Diskin SJ Zhang H Attiyeh EF Winter C Hou C. Integrative genomics identifies LMO1 as a neuroblastoma oncogene. Nature (2011) 469(7329):216–20. 10.1038/nature09609 PMC3320515 21124317

[33] Molenaar JJ Domingo-Fernandez R Ebus ME Lindner S Koster J Drabek K. LIN28B induces neuroblastoma and enhances MYCN levels via let-7 suppression. Nat Genet (2012) 44(11):1199–206. 10.1038/ng.2436 23042116

[34] Beckers A Van Peer G Carter DR Gartlgruber M Herrmann C Agarwal S. MYCN-driven regulatory mechanisms controlling LIN28B in neuroblastoma. Cancer Lett (2015) 366(1):123–32. 10.1016/j.canlet.2015.06.015 PMC4837470 26123663

[35] Schnepp RW Khurana P Attiyeh EF Raman P Chodosh SE Oldridge DA. A LIN28B-RAN-AURKA Signaling Network Promotes Neuroblastoma Tumorigenesis. Cancer Cell (2015) 28(5):599–609. 10.1016/j.ccell.2015.09.012 26481147 PMC4643330

[36] DuBois SG Marachelian A Fox E Kudgus RA Reid JM Groshen S. Phase I Study of the Aurora A Kinase Inhibitor Alisertib in Combination With Irinotecan and Temozolomide for Patients With Relapsed or Refractory Neuroblastoma: A NANT (New Approaches to Neuroblastoma Therapy) Trial. J Clin Oncol (2016) 34(12):1368–75. 10.1200/JCO.2015.65.4889 PMC4872349 26884555

[37] Gao L Lin P Chen P Gao RZ Yang H He Y. A novel risk signature that combines 10 long noncoding RNAs to predict neuroblastoma prognosis. J Cell Physiol (2019) 235(4):3823–34. 10.1002/jcp.29277 31612488

[38] Suo C Deng W Vu TN Li M Shi L Pawitan Y. Accumulation of potential driver genes with genomic alterations predicts survival of high-risk neuroblastoma patients. Biol Direct (2018) 13(1):1–11. 10.1186/s13062-018-0218-5 30012197 PMC6048860

[39] Formicola D Petrosino G Lasorsa VA Pignataro P Cimmino F Vetrella S. An 18 gene expression-based score classifier predicts the clinical outcome in stage 4 neuroblastoma. J Transl Med (2016) 14:142. 10.1186/s12967-016-0896-7 27188717 PMC4870777

[40] Zhang L Lv C Jin Y Cheng G Fu Y Yuan D. Deep Learning-Based Multi-Omics Data Integration Reveals Two Prognostic Subtypes in High-Risk Neuroblastoma. Front Genet (2018) 9:1–9. 10.3389/fgene.2018.00477 30405689 PMC6201709

[41] Wood AC Krytska K Ryles HT Infarinato NR Sano R Hansel TD. Dual ALK and CDK4/6 Inhibition Demonstrates Synergy against Neuroblastoma. Clin Cancer Res (2017) 23(11):2856–68. 10.1158/1078-0432.CCR-16-1114 PMC5457336 27986745

[42] Hart LS Rader J Raman P Batra V Russell MR Tsang M. Preclinical Therapeutic Synergy of MEK1/2 and CDK4/6 Inhibition in Neuroblastoma. Clin Cancer Res (2017) 23(7):1785–96. 10.1158/1078-0432.CCR-16-1131 27729458

[43] Geoerger B Bourdeaut F DuBois SG Fischer M Geller JI Gottardo NG. A Phase I Study of the CDK4/6 Inhibitor Ribociclib (LEE011) in Pediatric Patients with Malignant Rhabdoid Tumors, Neuroblastoma, and Other Solid Tumors. Clin Cancer Res (2017) 23(10):2433–41. 10.1158/1078-0432.CCR-16-2898 28432176

[44] Vallejo-Díaz J Chagoyen M Olazabal-Morán M González-García A Carrera AC. The Opposing Roles of PIK3R1/p85α and PIK3R2/p85β in Cancer. Trends Cancer (2019) 5(4):233–44. 10.1016/j.trecan.2019.02.009 30961830

[45] Fulda S. The PI3K_Akt_mTOR Pathway as Therapeutic Target in Neuroblastoma. Curr Cancer Drug Targets (2009) 9:729–37. 10.2174/156800909789271521 19754357

[46] González-Sancho JM García V Bonilla F Muñoz A. Thyroid hormone receptors_THR genes in human cancer. Cancer Letters (2003) 192:121–32. 10.1016/S0304-3835(02)00614-6 12668276

[47] Xiang Z Huang X Wang J Zhang J Ji J Yan R. Cross-Database Analysis Reveals Sensitive Biomarkers for Combined Therapy for ERBB2+ Gastric Cancer. Front Pharmacol (2018) 9:861. 10.3389/fphar.2018.00861 30123134 PMC6085474

[48] Raffaghello L Prigione I Airoldi I Camoriano M Levreri I Gambini C. Downregulation and/or release of NKG2D ligands as immune evasion strategy of human neuroblastoma. Neoplasia (2004) 6(5):558–68. 10.1593/neo.04316 PMC1531660 15548365

[49] Chi Y Wang D Wang J Yu W Yang J. Long Non-Coding RNA in the Pathogenesis of Cancers. Cells (2019) 8(9):1–44. 10.3390/cells8091015 PMC6770362 31480503

[50] Pandey GK Mitra S Subhash S Hertwig F Kanduri M Mishra K. The risk-associated long noncoding RNA NBAT-1 controls neuroblastoma progression by regulating cell proliferation and neuronal differentiation. Cancer Cell (2014) 26(5):722–37. 10.1016/j.ccell.2014.09.014 25517750

[51] Liu PY Tee AE Milazzo G Hannan KM Maag J Mondal S. The long noncoding RNA lncNB1 promotes tumorigenesis by interacting with ribosomal protein RPL35. Nat Commun (2019) 10(1):5026. 10.1038/s41467-019-12971-3 31690716 PMC6831662

[52] Thin KZ Liu X Feng X Raveendran S Tu JC. LncRNA-DANCR: A valuable cancer related long non-coding RNA for human cancers. Pathol Res Pract (2018) 214(6):801–05. 10.1016/j.prp.2018.04.003 29728310

[53] Yan Y Shi Q Yuan X Xue C Shen S He Y. DANCR_ an emerging therapeutic target for cancer. Am J Transl Res (2020) 12(7):4031–42. PMC7407722 32774756

[54] Meng X Li H Fang E Feng J Zhao X. Comparison of Stage 4 and Stage 4s Neuroblastoma Identifies Autophagy-Related Gene and LncRNA Signatures Associated With Prognosis. Front Oncol (2020) 10(1411):1–17. 10.3389/fonc.2020.01411 32974147 PMC7466450

[55] Utnes P Lokke C Flaegstad T Einvik C. Clinically Relevant Biomarker Discovery in High-Risk Recurrent Neuroblastoma. Cancer Inform (2019) 18:1176935119832910. 10.1177/1176935119832910 30886518 PMC6413431

[56] Sha M Lin M Wang J Ye J Xu J Xu N. Long non-coding RNA MIAT promotes gastric cancer growth and metastasis through regulation of miR-141/DDX5 pathway. J Exp Clin Cancer Res (2018) 37(1):58. 10.1186/s13046-018-0725-3 29540201 PMC5852965

[57] Liu Z Wang H Cai H Hong Y Li Y Su D. Long non-coding RNA MIAT promotes growth and metastasis of colorectal cancer cells through regulation of miR-132/Derlin-1 pathway. Cancer Cell Int (2018) 18:59. 10.1186/s12935-017-0477-8 29686537 PMC5902964

[58] Li Y Jiang B Wu X Huang Q Chen W Zhu H. Long non-coding RNA MIAT is estrogen-responsive and promotes estrogen-induced proliferation in ER-positive breast cancer cells. Biochem Biophys Res Commun (2018) 503(1):45–50. 10.1016/j.bbrc.2018.05.146 29792859

[59] Yang H Yang W Dai W Ma Y Zhang G. LINC00667 promotes the proliferation, migration, and pathological angiogenesis in non-small cell lung cancer through stabilizing VEGFA by EIF4A3. Cell Biol Int (2020) 44(8):1671–80. 10.1002/cbin.11361 32281700

[60] Yu J Wang F Zhang J Li J Chen X Han G. LINC00667/miR-449b-5p/YY1 axis promotes cell proliferation and migration in colorectal cancer. Cancer Cell Int (2020) 20:322. 10.1186/s12935-020-01377-7 32694944 PMC7368754

[61] Liu P Chen S Huang Y Xu S Song H Zhang W. LINC00667 promotes Wilms’ tumor metastasis and stemness by sponging miR-200b/c/429 family to regulate IKK-beta. Cell Biol Int (2020) 44(6):1382–93. 10.1002/cbin.11334 32129525

[62] Li J Song P Jiang T Dai D Wang H Sun J. Heat Shock Factor 1 Epigenetically Stimulates Glutaminase-1-Dependent mTOR Activation to Promote Colorectal Carcinogenesis. Mol Ther (2018) 26(7):1828–39. 10.1016/j.ymthe.2018.04.014 PMC6035735 29730197

[63] Zhong X Zhang Y Wang L Zhang H Liu H Liu Y. Cellular components in tumor microenvironment of neuroblastoma and the prognostic value. PeerJ (2019) 7:e8017. 10.7717/peerj.8017 31844563 PMC6910112

[64] Melaiu O Chierici M Lucarini V Jurman G Conti LA De Vito R. Cellular and gene signatures of tumor-infiltrating dendritic cells and natural-killer cells predict prognosis of neuroblastoma. Nat Commun (2020) 11(1):5992. 10.1038/s41467-020-19781-y 33239635 PMC7689423

