# Supplementary material for: Prognostic Signature of Immune Genes and Immune-Related LncRNAs in Neuroblastoma: A Study Based on GEO and TARGET Datasets
Source: Front Oncol. 2021 Mar 9;11:631546. doi: 10.3389/fonc.2021.631546 (PMC7985261; doi:10.3389/fonc.2021.631546)
Supplement: Supplementary file 1 [file Table_1.docx]

Supplementary Material

# Supplementary Data

## Supplementary Figures


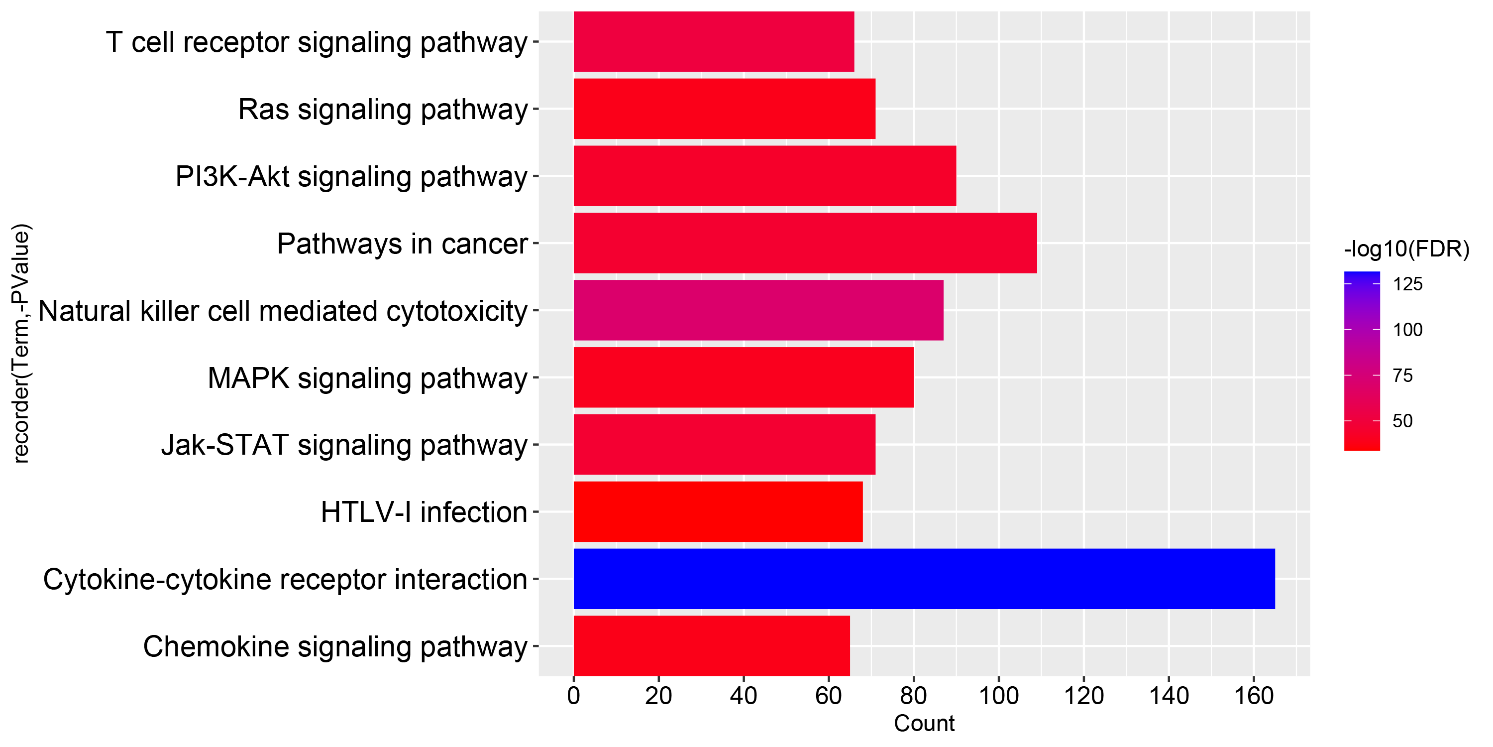


**Supplementary Figure S1**. KEGG pathways of prognostic immune-related genes.


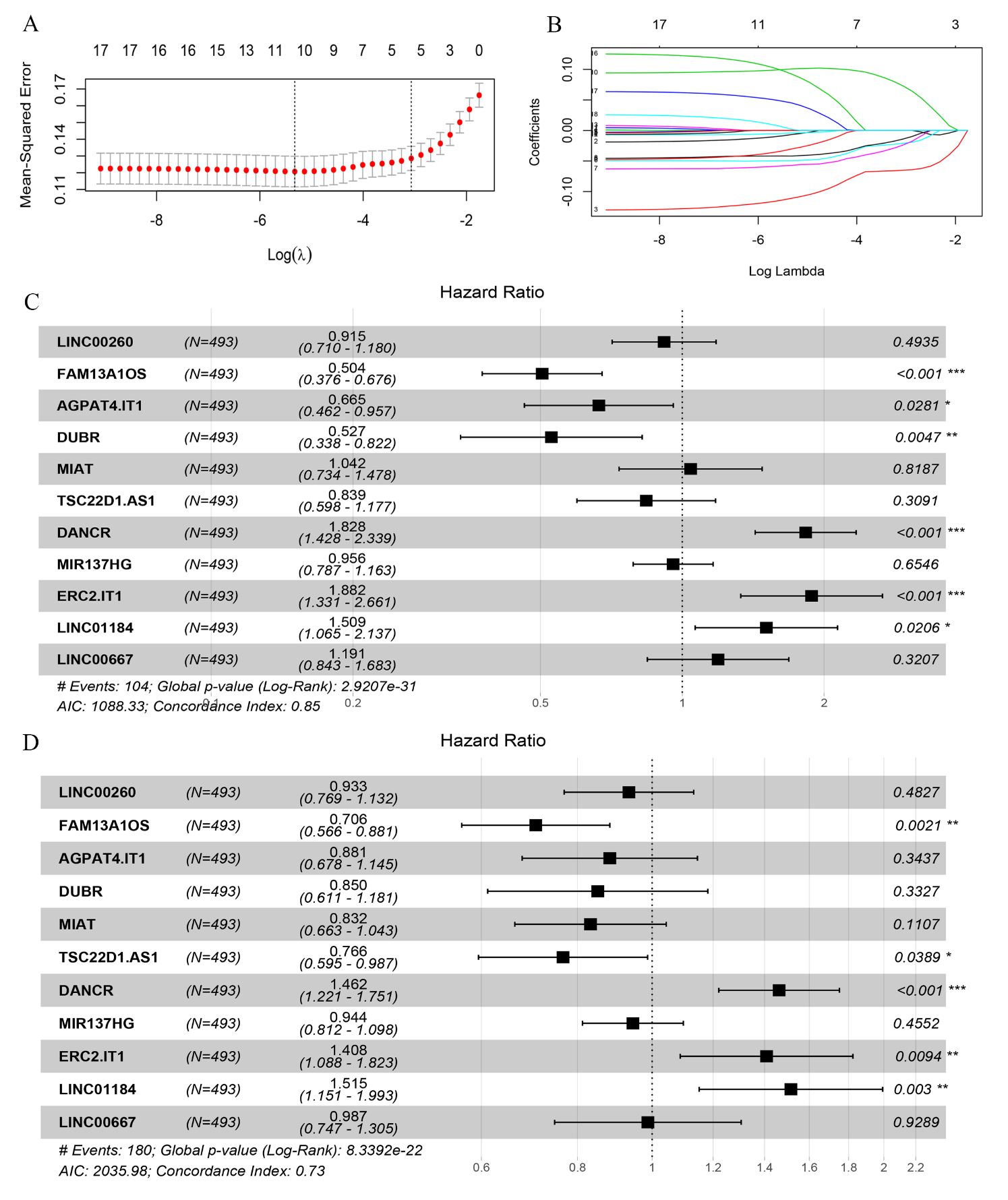


**Supplementary Figure S2.** Identification of immune-related lncRNAs. **(A, B)** LASSO Cox analysis identified eleven lncRNAs with the best prognostic value in GSE49710. **(C, D)** Multivariate Cox regression analysis of eleven lncRNAs for overall survival and event-free survival in GSE49710.


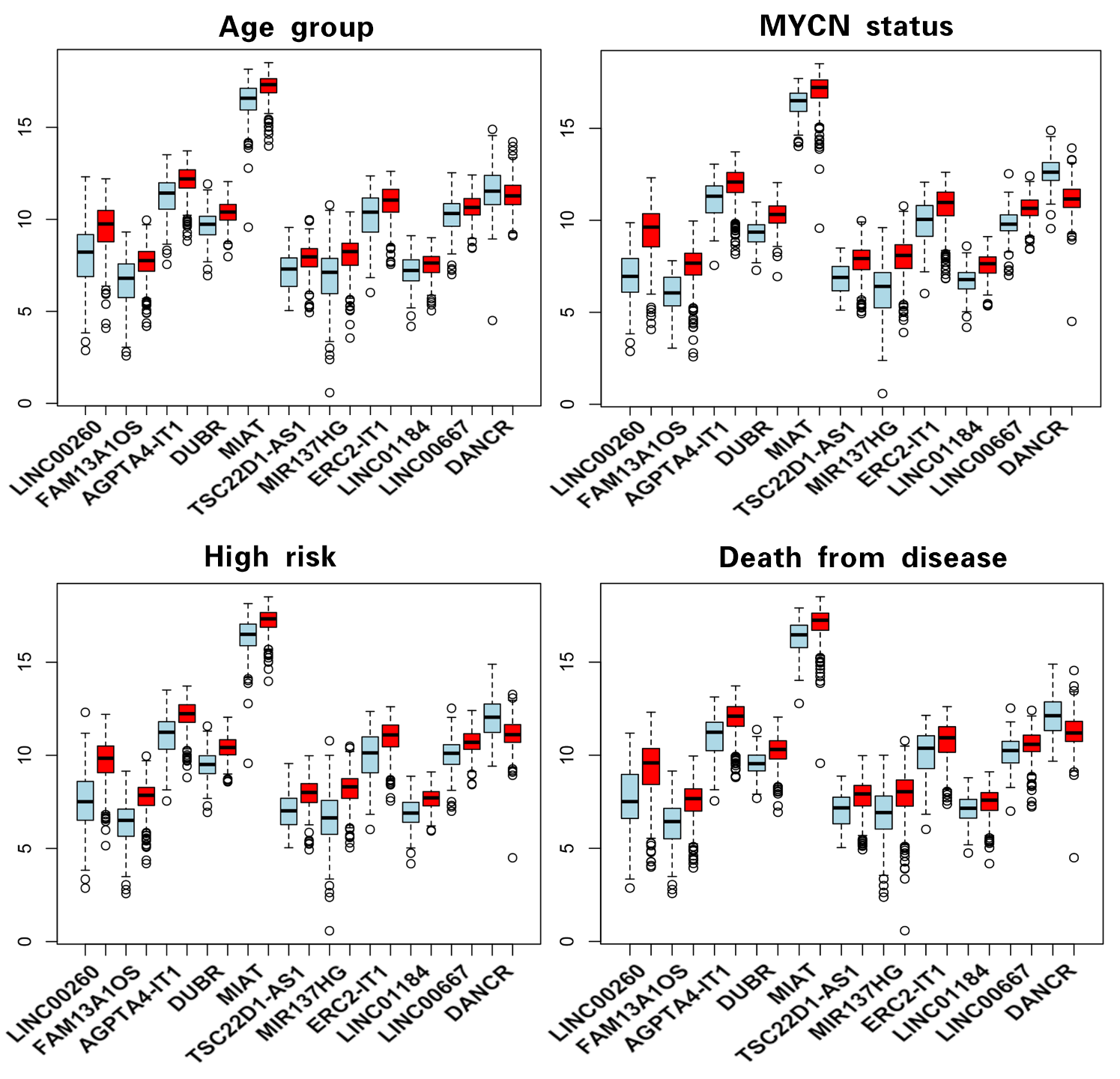


**Supplementary Figure S3.** LncRNAs in RS_Lnc were differentially expressed in different prognostic groups in GSE49710. Denotes: blue, age < 18 months, MYCN not amplified, not high risk and alive; red, age>= 18 months, MYCN amplified, high risk and death from disease. All p-values < 0.0001.

**
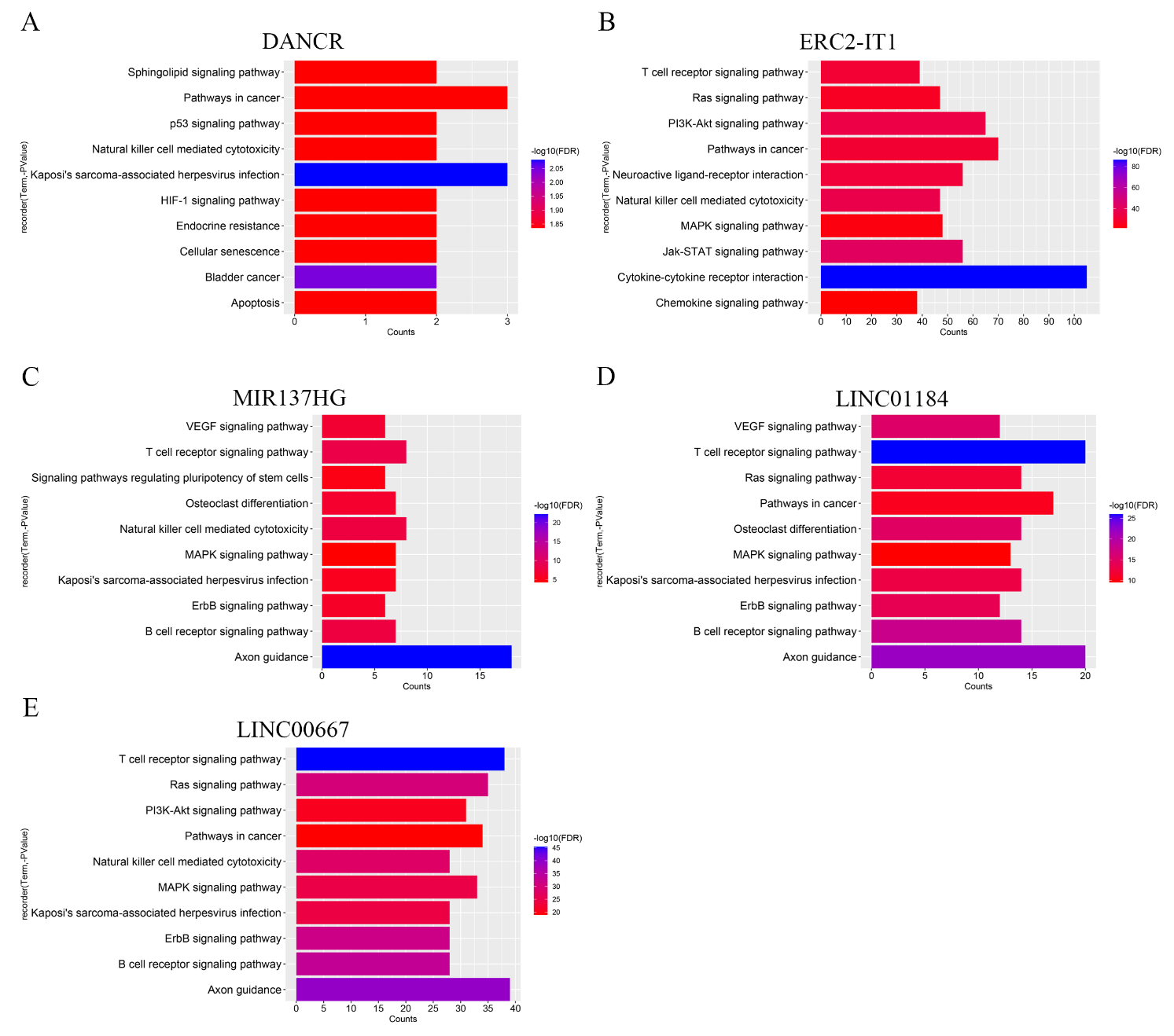
**

**Supplementary Figure S4**. KEGG pathway of immune-related lncRNAs.


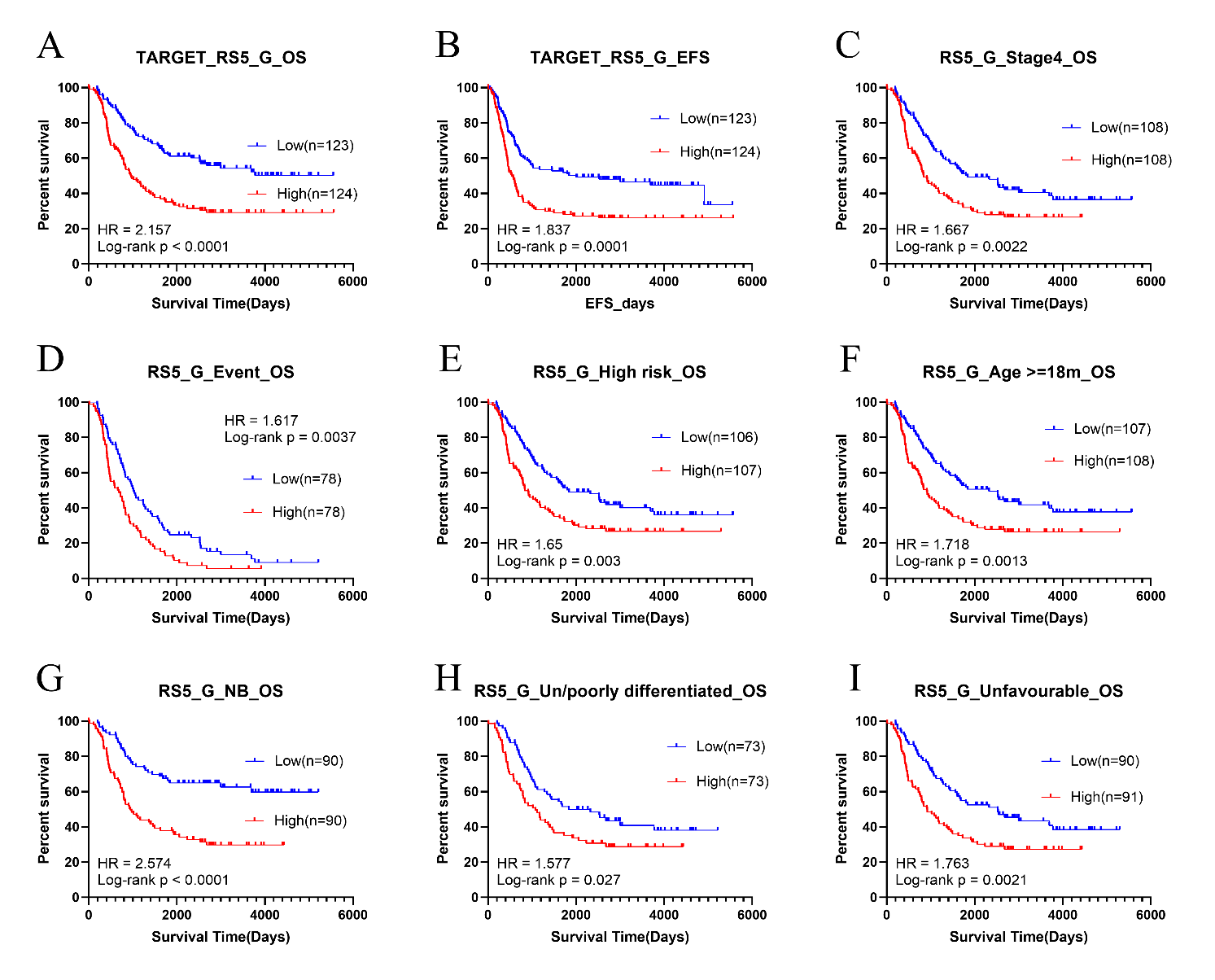


**Supplementary Figure S5.** Survival analysis of RS5_G in TARGET-NBL dataset. **(A, B)** Overall/event-free survival (OS/EFS) of high/low-risk score group. **(C-I)** Overall survival (OS) of patients with high/low-risk scores in subgroups.


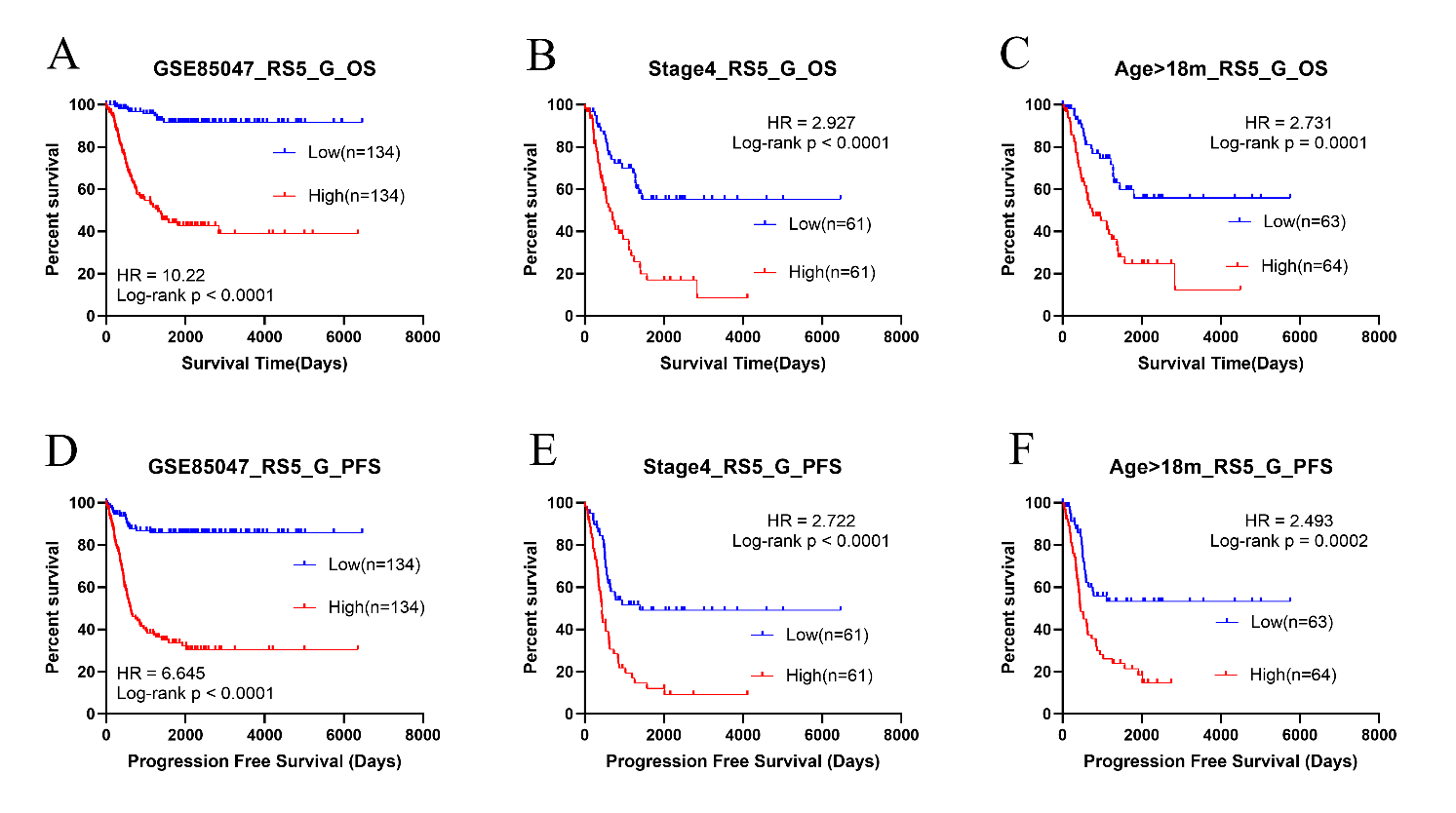


**Supplementary Figure S6.** Survival analysis of RS5_G in GSE85047 dataset. **(A-C)** Overall survival (OS) of high/low-risk score group in all patients and subgroups. **(D-F)** Progression-free survival (PFS) of patients with high/low-risk scores in all patients and subgroups.

## Supplementary Tables

**Supplementary Table S1.** Multivariate Cox Regression Analysis in high risk patients of GSE49710.

| Features | Overall Survival | | | Event-free Survival | | |
| --- | --- | --- | --- | --- | --- | --- |
|  | HR | 95% CI | p-value | HR | 95% CI | p-value |
| RS5_G | 1.759 | 1.416-2.186 | 3.35E-07 | 1.451 | 1.214-1.734 | 4.22E-05 |
| Age group |  |  | 0.646 |  |  | 0.666 |
| RS5_G | 1.726 | 1.362-2.187 | 6.16E-06 | 1.552 | 1.27-1.896 | 1.74E-05 |
| Mycn Amp |  |  | 0.739 |  |  | 0.157 |
| RS5_G | 1.747 | 1.407-2.169 | 4.21E-07 | 1.45 | 1.212-1.734 | 4.76E-05 |
| INSS_h1 |  |  | 0.207 |  |  | 0.343 |

HR: hazard ratio; CI: confident interval.

**Supplementary Table S2.** Multivariate Cox Regression Analysis in GSE49710.

| Features | Overall Survival | | | | Event-free Survival | | | |
| --- | --- | --- | --- | --- | --- | --- | --- | --- |
|  | HR | 95% CI | p-value | C-index | HR | 95% CI | p-value | C-index |
| RS_Lnc | 1.745 | 1.398-2.179 | 8.55E-07 | 0.871 | 1.475 | 1.248-1.743 | 5.21E-06 | 0.737 |
| Age group | 1.333 | 0.705-2.523 | 0.376 |  | 1.077 | 0.691-1.677 | 0.744 |  |
| High risk | 3.888 | 1.622-9.319 | 0.002 |  | 2.103 | 1.185-3.732 | 0.011 |  |
| Mycn Amp | 1.078 | 0.662-1.755 | 0.762 |  | 0.737 | 0.481-1.130 | 0.161 |  |
| INSS_h1 | 2.306 | 0.993-5.356 | 0.052 |  | 1.763 | 1.103-2.818 | 0.0178 |  |

**Supplementary Table S3.** Univariate Cox Regression Analysis in GSE16476.

| Features | Overall survival | | | |
| --- | --- | --- | --- | --- |
|  | HR | 95% CI | p-value | C-index |
| RS_Lnc | 2.718 | 2.025-3.648 | 2.71E-11 | 0.849 |
| Age group | 20.37 | 6.169-67.24 | 7.58E-07 | 0.779 |
| Sex | 0.906 | 0.451-1.822 | 0.783 | 0.518 |
| Mycn status | 5.225 | 2.589-10.54 | 3.94E-06 | 0.646 |
| INSS_h1 | 13.66 | 4.774-39.11 | 1.10E-06 | 0.756 |
| RS5_G | 2.718 | 2.04-3.621 | 8.40E-12 | 0.852 |

**Supplementary Table S4.** Multivariate Cox Regression Analysis in GSE16476.

| Features | HR | 95% CI | p-value | C-index | HR | 95% CI | p-value | C-index |
| --- | --- | --- | --- | --- | --- | --- | --- | --- |
| RS_Lnc | 1.946 | 1.372-2.759 | 1.87E-04 | 0.882 | NA | NA | NA | NA |
| Age group | 5.823 | 1.384-24.506 | 0.0163 |  | 8.421 | 2.106-33.68 | 2.59E-03 | 0.883 |
| Sex | 1.031 | 0.498-2.137 | 0.934 |  | 1.141 | 0.55-2.366 | 0.723 |  |
| Mycn status | 0.736 | 0.336-1.613 | 0.444 |  | 1.042 | 0.475-2.288 | 0.918 |  |
| INSS_h1 | 2.067 | 0.564-7.57 | 0.273 |  | 1.731 | 0.486-6.164 | 0.397 |  |
| RS5_G | NA | NA | NA | NA | 2.069 | 1.46-2.934 | 4.45E-05 |  |

**Supplementary Table S5.** Univariate Cox Regression Analysis in GSE85047.

| Features | Overall Survival | | | | Progression-free Survival | | | |
| --- | --- | --- | --- | --- | --- | --- | --- | --- |
|  | HR | 95% CI | p-value | C-index | HR | 95% CI | p-value | C-index |
| RS5_G | 2.718 | 2.184-3.384 | <2E-16 | 0.793 | 2.34 | 1.937-2.827 | <2E-16 | 0.749 |
| Age group | 6.978 | 3.874-12.57 | 9.70E-11 | 0.691 | 4.171 | 2.649-6.568 | 7.05E-10 | 0.645 |
| Mycn status | 5.806 | 3.633-9.278 | 1.95E-13 | 0.676 | 3.886 | 2.575-5.866 | 1.04E-10 | 0.628 |
| INSS_h1 | 22.22 | 6.985-70.67 | 1.50E-07 | 0.711 | 7.005 | 3.729-13.16 | 1.43E-09 | 0.659 |

**Supplementary Table S6.** Multivariate Cox Regression Analysis in GSE85047.

| Features | Overall Survival | | | | Progression-free Survival | | | |
| --- | --- | --- | --- | --- | --- | --- | --- | --- |
|  | HR | 95% CI | p-value | C-index | HR | 95% CI | p-value | C-index |
| RS5_G | 1.915 | 1.422-2.579 | 1.86E-05 | 0.835 | 1.814 | 1.413-2.329 | 2.92E-06 | 0.765 |
| Age group | 1.893 | 0.973-3.681 | 0.06 |  | 1.441 | 0.834-2.49 | 0.19 |  |
| Mycn status | 1.638 | 0.981-2.737 | 0.059 |  | 1.298 | 0.818-2.059 | 0.27 |  |
| INSS_h1 | 7.381 | 2.138-25.482 | 0.00157 |  | 2.993 | 1.442-6.212 | 3.25E-03 |  |
